# Can longitudinal generalized estimating equation models distinguish network influence and homophily? An agent-based modeling approach to measurement characteristics

**DOI:** 10.1186/s12874-016-0274-4

**Published:** 2016-12-28

**Authors:** Kori Sauser Zachrison, Theodore J. Iwashyna, Achamyeleh Gebremariam, Meghan Hutchins, Joyce M Lee

**Affiliations:** 1 Department of Emergency Medicine, Massachusetts General Hospital and Harvard Medical School, Zero Emerson Place Suite 3B, 55 Fruit Street, Boston, MA 02114 USA; 2 VA HSR&D Center of Excellence, Ann Arbor, MI USA; 3 Department of Internal Medicine, University of Michigan, Ann Arbor, MI USA; 4 Division of Pediatric Endocrinology, Child Health Evaluation and Research Unit (CHEAR), Department of Pediatrics, University of Michigan, Ann Arbor, MI USA; 5 Center for the Study of Complex Systems, University of Michigan, Ann Arbor, MI USA

**Keywords:** Network Analysis, GEE Models, Network Influence

## Abstract

**Background:**

Connected individuals (or nodes) in a network are more likely to be similar than two randomly selected nodes due to homophily and/or network influence. Distinguishing between these two influences is an important goal in network analysis, and generalized estimating equation (GEE) analyses of longitudinal dyadic network data are an attractive approach. It is not known to what extent such regressions can accurately extract underlying data generating processes. Therefore our primary objective is to determine to what extent, and under what conditions, does the GEE-approach recreate the actual dynamics in an agent-based model.

**Methods:**

We generated simulated cohorts with pre-specified network characteristics and attachments in both static and dynamic networks, and we varied the presence of homophily and network influence. We then used statistical regression and examined the GEE model performance in each cohort to determine whether the model was able to detect the presence of homophily and network influence.

**Results:**

In cohorts with both static and dynamic networks, we find that the GEE models have excellent sensitivity and reasonable specificity for determining the presence or absence of network influence, but little ability to distinguish whether or not homophily is present.

**Conclusions:**

The GEE models are a valuable tool to examine for the presence of network influence in longitudinal data, but are quite limited with respect to homophily.

**Electronic supplementary material:**

The online version of this article (doi:10.1186/s12874-016-0274-4) contains supplementary material, which is available to authorized users.

## Background

A ubiquitous feature of networks is that nodes connected by a relationship are more likely to share a given salient attribute than are two randomly selected nodes. This cross-sectional finding may arise from at least two mechanisms: either the presence of the relationship may make the nodes change their attribute so as to become more alike, or nodes that are more alike may be more likely to form a relationship. The former mechanism may be termed network influence, the latter homophily. A crucial question in empirical network analysis has been to distinguish these two mechanisms, and from a third possible mechanism, that of shared context.

The ability to distinguish between network influence and homophily is critical in various contextual applications of social network analysis. Certainly, the application in the relationship between obesity and formation of friendship connections has been well-described and will be the primary example carried through this report. But this distinction between network influence and homophily is broadly relevant. For example, we may consider a network composed of many hospitals (nodes) that are connected through the transfer of patients from smaller less-resourced hospitals to bigger tertiary care hospitals. Such a network may be built entirely by homophily where hospitals partner based on similarity of practice patterns. In such a case, shaping the network is unlikely to result in dissemination of best practice. Without the omniscience to observe and understand these dynamics from the onset, we are dependent upon other methods for distinguishing between network influence and homophily.

The analyses of the social network of the Framingham Study by Christakis and Fowler gave high visibility to one statistical approach to distinguishing network influence in longitudinal data [1, 2]. They used a generalized estimating equations (GEE) framework, taking the dyad as the unit of analysis, and using multi-level modeling to account for the non-independence of observations around any given ego. Certainly there are other approaches to this question—particularly actor-oriented models (such as are operationalized in SIENA [3]), dynamic propensity-score matching [4], the diffusion of innovation traditions [5, 6], or the use of instrumental variables [7, 8]. However, the GEE approach is readily accessible to many non-network social scientists, can be implemented in the conventional statistical software already used for other purposes, and seems to be estimatable in large empirical networks [9]. As such, the GEE approach warrants close examination.

To what extent does the GEE-based approach accurately distinguish network influence and homophily? The approach has been vigorously discussed on a number of grounds, as reviewed in the next section. The contribution of the present manuscript is different. We used an agent-based model (ABM) to simulate the collection of data in a cohort study. Within this agent-based model, the full extent of network influence and homophily could be known—they were programmed into the model. Data from the ABM were harvested and subjected to GEE-based analysis as if they had been cohort data. Our core question is: to what extent, and under what conditions, does the GEE-approach recreate the actual dynamics present in the generating ABM?

### Past literature on GEE-based approaches to network influence

The empirical work on the network spread of obesity achieved high visibility in both the scientific and lay press. The work has been subjected to a variety of critiques, none convincing to the entire community. For example, Cohen-Cole and Fletcher analyzed data from Add Health to examine the extent of network influence in rates of self-reported acne and headaches [10]. They found that adding school-level fixed effects attenuated the apparent network influence effects towards zero, and removed their statistical significance at conventional levels. In a further analysis, Cohen-Cole and Fletcher demonstrated that failing to account for the auto-correlation in one‘s own height over time can also lead to spurious inference of network influence. However, as these applications hinge on re-analysis of a defined empirically collected data set—in which the true data generating process is, of necessity, unknown—it remains a question as to how relevant to empirical analyses these critiques are. It might also be argued that auto-correlation, collinearity, and shared environment are not unique to networks, but are instead ubiquitous features of longitudinal modeling in social science. In a special section of the *Journal of Health Economics*, several authors extended these concerns in explorations of Add Health, with different combinations of waves, fixed effects, and parameterizations of the dependent variable—leading to the suggestion that the replicability of the Framingham data in Add Health data might be specification-dependent [2, 11, 12]. More recently, similar results have been obtained from stochastic actor-oriented models, implemented in SIENA [13].

Two additional papers have issued wide-ranging critiques of the GEE model, both making very strong claims on conceptual grounds. Shalizi and Thomas argue that “latent homophily and contagion are generically confounded with each other … and any direct contagion effects cannot be nonparametrically identified from observational data,” and that strong parametric assumptions are needed unless quite strong assumptions can be made about the structure of the data generating process on substantive grounds [14]. Lyons has severely critiqued nearly every aspect of the Christakis & Fowler paper, arguing that the basic modeling assumptions of the GEE suggest that it cannot be used in the network context [15]. Yet VanderWeele et al. have demonstrated that in fact, Lyons’ concerns of model inconsistency and statistical dependence can be solved when testing for contagion [16].

The goal of the present manuscript is not to directly intervene in these theoretical arguments, which have spilled over to the popular press [17]. Rather, we note that there also exists a large body of research suggesting that even when strict regression assumptions are violated, the analysis may still be useful [18, 19]—a crucial question from this perspective is the extent to which the GEE model’s interpretation is sensitive to these violations.

Moving in that direction, Noel and Nyhan examined the robustness of the GEE analyses in a different approach, using simulation-based models [20]. They examined the extent to which the breaking of network ties could lead to bias in apparent network influence. Their examination showed that if more homophilous ties are likely to be more enduring, then this could lead to spurious inference of network influence. This important work highlighted the potential value of a simulation-based approach for establishing the measurement characteristics of the GEE-based analysis of networks, but did not address the fundamental extent to which the GEE-based models could distinguish homophily from network influence. Therefore our primary objective is to determine to what extent, and under what conditions, does the GEE-approach recreate the actual dynamics in an agent-based model and to what extent can longitudinal GEE models distinguish network influence and homophily.

## Methods

### Analytic approach

Our approach to quantifying the GEE model performance is to consider the statistical regression as a test from the perspective of measurement theory. Any test has a sensitivity (the probability of a positive test result being obtained when, in fact, the positive condition holds) and a specificity (the probability of a negative test result being obtained when, in fact, the negative condition holds). Further, this data can be formulated as a likelihood ratio to allow Bayesian updating in light of a test result: given one’s prior odds about whether or not network influence (for example) was present, to what extent should a regression indicating the presence of network influence increase those odds? The positive likelihood ratio is calculated as (Sensitivity/(1—Specificity) ; the negative likelihood ratio is (1—Sensitivity)/Specificity. Posterior odds after having seen the regression result are the prior odds multiplied by the appropriate likelihood ratio. These are related to the familiar concepts of Type I and Type II error, but more explicitly oriented towards understanding the extent to which new empirical results should change one’s prior beliefs about the way the world worked. We further ask the secondary question: when network influence is present in the underlying model, are the GEE parameters sensitive to changes in the strength of that network influence?

It is important to note that the calculation of sensitivity and specificity depend on the dichotomization of the test statistic into a positive or negative. For the purposes of this analysis, we follow Christakis and Fowler and define affirmative evidence as a regression coefficient with a *p-*value of 0.05 or below. This implies that we might expect specificity of 0.95 under a well-performing regression—that 5% of runs where there is no effect, a regression will nonetheless be “statistically significant” at the *p <* 0.05 level.

We considered the performance of the GEE model in several cases. Our substantive interest was in network influence and homophily in adolescent obesity. As such, we generated populations of simulated cohorts where either, both, or neither network influence and homophily may occur. All simulated populations were gradually gaining weight, consistent with observed secular trends. We then determined the sensitivity, specificity and likelihood ratios for GEE regression results in these populations. We considered models that do and do not control for these secular trends. In addition to the results here, we have posted the ABM-generating code at an archive site, so that others wishing to ascertain the measurement characteristics of alternative empirical approaches can do so with ease—replicating or extending these results.

### Generating populations for simulated cohort studies

Our goal with the ABM was to develop a flexible simulation code for simulating populations as if they were in a cohort study. Each cohort member is simulated using a separate agent. At the beginning of the simulation, each agent has a baseline weight (drawn from a uniform distribution tunable with respect to a set minimum (80lbs) and maximum (300lbs) weight) and an intrinsic rate of weight gain (drawn from a uniform distribution tunable with respect to minimum (0.0lbs) and maximum (2.0lbs) intrinsic gain per month). By “tunable”, we mean a value can be set by the analyst for each simulation run; values are fixed at the beginning of the simulation and for the entire cohort simulation process. In an extension of the model, we had patients draw from a normal distribution of weights with a mean of 190 and a standard deviation of 70, to test if our results were sensitive to the shapes of these parameters.

Each agent also has the capacity to designate a tunable number of friends, which can be fixed (we fix ours at 1). Consistent with observed behavior of adolescents, friend nominations need not be reciprocated. In simulations in which homophily is not present, the selection of friends is done without respect to characteristics of the friend, by choosing at random from other members of the population of agents. In simulations in which homophily is to be present, first the absolute difference between the agent (ego) and all other agents (alters) is calculated. In the base case, each alters’ probability of being chosen as a friend is proportional to the reciprocal of the weight difference; the extent to which weight difference is important to the choice of friends is tunable for each run of the model. In extensions of the model, we include preferential attachment in the choice of friends, using the algorithm described by Newman [21]. Preferential attachment is tested with and without homophily on the basis of weight. In these cases, the probability of being selected as a friend is greater for nodes that already have more friends, in addition to any homophily effects.

We consider static simulations, in which the friendship networks are formed in the first period and do not change thereafter. (Such a situation applies when the rate of change of network ties is slow relative to the time-span under observation in the study, not merely to truly “static” networks for the entire life of the network.) We also consider dynamic networks, in which friendship networks change at tunable intervals (we set ours to every 30 time-steps).

In simulations in which network influence is present, each agent’s actual weight gain is a tunably weighted average of their own intrinsic weight gain rate and the difference between their current weight and the weight of their friends. Weight gain can be negative, if a given agent is friends with much lighter agents.

Having established the basis for each agent, a population of agents is then created and given initial values. At each simulation time-step, the agents’ weights change, including any network influences as specified by the parameters. In dynamic network models, friend nominations are made only after all agents’ weights have been updated. Agents are activated in a random order each time-step, but all are activated once and only once per round. Agents do not enter or exit the model during a run. After a user-defined number of time-steps (120 for us), a network data set is output, enumerating each agent, their current weight, and their current outgoing friend nominations at each time-step.

Initial values for parameters in the present case were set to model a potential study of weight gain in the U.S. Thus for the base case, we set each cohort size to 30; set a minimum intrinsic weight gain of 0.0 lbs and a maximum of 2.0 lbs pounds per time-step (“month”); each agent had one friend; and simulated cohort data collection were output for statistical analysis for each time-step (simulated month). In extensions, we replicated with cohort sizes of 1000 to test the extent to which the GEE’s performance varied across a range of feasible study sizes.

While we have explained this model in terms of weight and friendship, these models are not constructed so as to closely mimic physiology or some other application-specific characteristic. The agents, in fact, simply have one continuous-valued attribute with an intrinsic rate of growth of that attribute (which may be mean zero), and a propensity to develop relationships with other agents that may be based on that attribute.

This initial ABM model intentionally did not feature several additional complications that might be present in the real world—it was designed to examine the baseline performance of the GEE approach. Real world data would include missing data, random variation in weight gain and measurement of weight, heterogenous and variable numbers of friends, and other such complications in the data generating process. Our framework is readily extensible to such conditions, but for clarity they were not included in this first examination.

### Statistical analysis using GEE

We simulated the collection of cohort data by examining the characteristics of agents and their network structure as a subset of time-steps—in our case, analyzing data from time-steps 24, 48, 72, 96, and 120 (as if biennial data collection). Since each analysis required lagged values, data from time-step 24 was used only to produce those lagged values.

The basic analytic framework estimated a dyadic-level GEE. The unit of analysis was an ego-alter pair for each wave of the survey, with the current and lagged weights of the ego and alter. Egos could appear multiple times in each survey wave, once with each alter to whom he or she was paired. Ties that had dissolved no longer contributed data. The following basic form was used for the estimation: (with subscripts indicating contemporaneous and lagged variable measurement, and regression coefficients suppressed for clarity).

$$ \mathrm{Ego}\ \mathrm{Weigh}{\mathrm{t}}_{\mathrm{T}} = \mathrm{Alter}\ \mathrm{Weigh}{\mathrm{t}}_{\mathrm{T}} + \mathrm{Ego}\ \mathrm{Weigh}{\mathrm{t}}_{\mathrm{T}\hbox{-} 1} + \mathrm{Alter}\ \mathrm{Weigh}{\mathrm{t}}_{\mathrm{T}\hbox{-} 1}+\upxi $$

These terms were interpreted as follows, following Christakis and Fowler [1, 2]. The coefficient on Alter Weight _T_ was interpreted as the evidence of network influence. Alter Weight _T-1_ was interpreted as the homophily parameter. Ego Weight _T-1_ was interpreted as controlling for genetic endowments and the past history of the ego.

An exchangeable error structure was used in the GEE model to adjust the standard errors for the non-independence of Ego observations. Christakis and Fowler reported that, in general, their results were not sensitive to the particular form of the error structure that was specified [1]. In the original article on the network spread of obesity, weight was incorporated as a dichotomous variable for obese or not. In our analyses we used continuous variables.

We automated the process of analysis using Stata 10 [22]. Each data set was opened, GEE regressions were run, and coefficients were stored in a summary dataset for further analysis. As is conventional, we considered *p <* 0.05 statistically significant, and focused on the presence or absence of statistically significant findings rather than on the magnitude of the coefficients. Unless otherwise specified, all regressions controlled for the “survey wave” using a vector of indicator variables.

### Availability for replication and extension

The following items are available, permanently archived at the Dryad Digital Repository (http://dx.doi.org/10.5061/dryad.v3s0k): the ABM generating code; each of the populations of simulated cohorts analyzed in this manuscript; and the Stata code used to implement the GEE model.

## Results

We examined the performance of the regression under a number of simulation conditions. In Table 1, we show the basic results from a series of simulation runs and analyses under the conditions where homophily was set to zero and the network was static. In runs when network influence was present in the ABM, it was always detected by the regression, yielding a sensitivity of 100%. In runs where network influence was set to zero in the ABM, regressions had significant coefficients for network influence 9.1% of the time (by chance this would be expected 5%), giving a specificity of 90.9%. These corresponded to positive likelihood ratios of 11.0 and a negative likelihood ratio of 0.

**Table 1:** GEE Evidence of Network Influence, ABM with No Homophily on Observed Weight

	Did ABM Have Network Influence?	
Regression Coefficient For Network Influence	Yes	No
Significant	1000	91
Not Significant	0	909

A full set of measurement parameters are shown in Table 2, 3, 4 and 5 for static networks. The GEE displayed reasonable ability to distinguish network influence, regardless of homophily. Absent homophily, the sensitivity for network influence was 100% and the specificity was 90.9%, as presented above (Table 2). When homophily was present, the sensitivity for network influence was 100%, and the specificity was 90.5%. As shown in Table 3, similar results were obtained when friendships displayed preferential attachment, and when the cohort size for each model run was increased to 1000 from 30. In all of these extensions, weights were drawn from a normal distribution rather than a uniform distribution.

**Table 2:** Measurement Characteristics from Static Networks: Testing for Network Influence, without and with possible confounding by homophily

	Cohort = 30	No Preferential Attachment
	Homophily Absent	Homophily Present
Sensitivity	100%	100%
Specificity	91%	91%
LR+	11.0	10.5
LR-	0	0

(2000 ABM model runs per column)

**Table 3:** Measurement Characteristics from Static Networks: Testing for Network Influence in Cases with Preferential Attachment (200 ABM model runs per column)

	Cohort = 30	Preferential Attachment	Cohort = 1000	Preferential Attachment
	Homophily Absent	Homophily Present	Homophily Absent	Homophily Present
Sensitivity	100%	100%	100%	100%
Specificity	93%	95%	92%	97%
LR+	14.3	20.0	12.5	33.3
LR-	0	0	0	0

**Table 4:** Measurement Characteristics from Static Networks: Testing for Homophily on Observed Weight, without and with possible confounding by network influence

	Cohort = 30	No Preferential Attachment
	Network Influence Absent	Network Influence Present
Sensitivity	9.5%	100%
Specificity	91%	0%
LR+	1.04	1.00
LR-	1.00	1.00

(2000 ABM model runs per column)

**Table 5:** Measurement Characteristics from Static Networks: Testing for Homophily in Cases with Preferential Attachment (200 ABM model runs per column)

	Cohort = 30	Preferential Attachment	Cohort = 1000	Preferential Attachment
	Network Influence Absent	Network Influence Present	Network Influence Absent	Network Influence Present
Sensitivity	5.0%	100%	3.0%	100%
Specificity	0%	0%	92%	0%
LR+	0.05	1.00	0.38	1.0
LR-	1.0	1.00	1.05	1.0

In contrast, the GEE was unable to distinguish situations in which homophily was present in the original friend formation of the static network from ABM runs where there was no homophily. (Table 4) If network influence was absent, then the homophily coefficients were statistically significant in 9.5% of cohorts in which homophily was present, and in 9.1% of cohorts in which homophily was absent, yielding a sensitivity of 9.5% and specificity of 90.9%. If network influence was present in the ABM, then homophily coefficients were positive in 100% of regressions, regardless of whether or not homophily was present in the ABM. Thus, for the purposes of distinguishing homophily, all likelihood ratios were approximately 1, indicating that these regression results provide no additional information to change one’s prior belief about the presence of homophily. Table 5 demonstrates that there was little change in the measurement characteristics when simulations were run changing the distribution of friendships to include preferential attachment, drawing initial weights from a normal rather than uniform distribution, and changing the cohort size for each ABM model run to 1000.

Of note, our parameters were set with a non-zero mean weight gain for the population as a whole, consistent with our interest in the current U.S. setting. In that context, we found that if the GEE model was estimated without controls for survey wave, then the network influence parameters were always positive, even in runs in which neither network influence nor homophily were present. Thus all analyzed models here control for survey wave as a series of indicator variables, and this is likely essential to effective estimation in a real application.

We further considered situations in dynamic networks, where agents could reform friendships every 30 time-steps. Thus friendship networks reformed 4 times during the simulated longitudinal cohort, somewhat less frequently than the 5 periodic data collections provided to the regressions. We find a very similar pattern of measurement characteristics. In situations where network influence was present in the ABM, it was detected with 100% sensitivity and 93% specificity regardless of whether or not there was possible confounding by homophily—corresponding to positive likelihood ratios of greater than 14, and negative likelihood ratios of 0. (See Table 6) In contrast, the regressions were poor at distinguishing whether or not homophily was present—all likelihood ratios were approximately 1.0 in these models where friendships reformed several times during the simulated data collection (See Table 7).

**Table 6:** Measurement Characteristics from Dynamic Networks: Testing for Network Influence, without and with possible confounding by homophily

	Homophily Absent	Homophily Present
Sensitivity	100%	100%
Specificity	93.2%	93.1%
LR+	14.7	14.5
LR-	0	0

(Cohort size = 30, no preferential attachment, 2000 ABM model runs per column)

**Table 7:** Measurement Characteristics from Dynamic Networks: Testing for Homophily on Observed Weight, without and with possible confounding by network influence

	Network Influence Absent	Network Influence Present
Sensitivity	8.7%	100%
Specificity	92.4%	0%
LR+	1.14	1.00
LR-	0.99	1.00

(Cohort size = 30, no preferential attachment, 2000 ABM model runs per column)

We tested for the empirical importance of an additional potential threat to the validity of the network influence regressions. (Table 8) In this case, homophily occurred on the basis not of realized weight, but on the basis of intrinsic weight gain. This corresponds to a situation in which individuals make friends on the basis of a shared affinity for physical activity (or entertainment lacking physical activity), what Shalizi and Thomas termed “latent homophily” [14]. Pertinently, in many situations such affinities may not be observable. Thus, in this case, homophily occurred on the basis of intrinsic weight gain, but such data was not available to regressions. In the dynamic case, the GEE model was able to distinguish situations where network influence (on the basis of realized weight) was present with a sensitivity of 100%, a specificity of 93.0%, yielding a positive likelihood ratio of 14.3 and a negative likelihood ratio of 0. Measurement characteristics for the static network were quite similar.

**Table 8:** Measurement Characteristics in the Presence of Homophily on Unobservable Intrinsic Weight Gain Rather than Observed Weight: Testing for Network Influence, in the presence of possible confounding by homophily on unobserved intrinsic rate of change

	Static Network	Dynamic Network
Sensitivity	100%	100%
Specificity	91.5%	93.0%
LR+	11.8	14.3
LR-	0	0

(Cohort size = 30, no preferential attachment, 2000 ABM model runs per column)

Finally, we asked whether the network influence coefficients were responsive to changes in the importance of network influence in the ABM model. The ABM models were designed to be behaviorally plausible and to have network interactions, rather than to mimic the structure of a GEE, so there is not a simple answer as to what the “right” network influence coefficient should be in the GEE scale. We considered the static model with cohort size 30, network influence, initial weights from a normal distribution, and preferential attachment—with no homophily and no latent homophily. In the initial models the mean coefficient for network influence was 0.1217 (standard deviation: 0.0018) with a median of 0.1216. As a test, we doubled the network influence parameter in the ABM models on a tick-to-tick basis. Rerunning the GEE models showed a mean for network influence of 0.2320 (standard deviation: 0.0076) with a median of 0.2313.

## Discussion

Our results demonstrate that some caution should be used when using GEE-based frameworks to test for the presence of network influence and homophily in longitudinal networks. For network influence, we find that the approach appears to have excellent sensitivity, and quite good specificity with regard to distinguishing the presence or absence of such a “network effect”, regardless of whether or not homophily is present in network formation. This was true for small cohorts (*n =* 30) and larger cohorts (*n =* 1000), and for cohorts that displayed lesser and greater realism in their distribution of friendships. The *p-*values from the GEE models for network influence may overstate significance, and in practice corrections (e.g., the Kauermann and Carroll correction) should be utilized [23]. Further, when network influence was present, the GEE network influence coefficient was responsive to changes in the underlying ABM behavioral dynamics. In contrast, the models show little ability to distinguish when homophily is present from when it is absent.

Beyond these concrete results, our further contribution is to provide a clear, readily extensible framework in which to pragmatically test identification and bias claims in a population in which the underlying data generating mechanism is known with certainty. By comparing the GEE-based estimates against an agent-based model, we improve upon past literature by providing an extensible framework within which possible confounding can be tested. The underlying ABM framework allows detailed, methodologically individual specifications of behavioral interaction rules. These can then be presented to a proposed analytic approach to verify its robustness. Given the code already implemented, it should be relatively straightforward to check additional complications. Further, our baseline data sets are archived and readily available for testing. The results in Table 8 provide an example of the potential value of this approach testing estimation strategies. Homophily on the presence of unobservable shared tastes for activity could not be directly observed, by definition. As such, the extent to which empirically derived results might be confounded could only be argued for or against, not tested in a human population, whereas it can be tested in an agent-based population.

These results suggest that the GEE models provide an important tool to the analytic armamentarium to examining for the presence of network influence in longitudinal data. Aral has recently argued for a careful definition of exactly what processes are included in the umbrella “network influence” [24]. He demonstrates that theoretical clarity about potential causal mechanisms might imply different empirical specifications, extending classic debates about the relative importance of cohesion and equivalence [25]. The present work takes a complementary tack, focusing on the extent to which network influence—however appropriately theoretically specified—can be distinguished by a given empirical approach. Our results further suggest that the GEE-based models are able to distinguish network influence from its absence even when there is a more subtle homophily—so-called latent homophily [14] on an unobservable intrinsic parameter for rate of change, rather than the observed characteristic itself.

For many scholars, empirical evidence of causal network influence is the key analytic goal; but for others, homophily may be an affirmative object of interest, rather than merely a confounder that impedes adequate detection of causal network influence. For example, Moody has compellingly re-formulated classic sociological concerns about group-level racial integration in terms of micro-dynamics of friendship-level racial homophily [26]. More generally, the role of intergroup mixing and homophily are subjects of broad relevance to understanding many social problems. [27] Our results suggest that the GEE-based models provide little evidence of value as to whether or not such homophily has been present in explaining longitudinal data patterns. (It is important to note that Christakis and Fowler never claim to substantively interpret the “homophily” parameter—this is simply an obvious, but wrong, generalization of their explanations for their analytic strategy.) Actor-oriented stochastic models may be more appropriate for exploring homophily effects in dynamic networks [3, 13].

These results have several important limitations. Most importantly, the ABMs have a number of independently tunable parameters. We have examined GEE performance for a series of those parameters that align with one substantive interest. An exhaustive search of the parameter space is infeasible, but the GEE models may have different measurement characteristics in other regions of that space. As such, we have made the generating models available so that others may replicate these analyses in the parameter space most relevant to their particular proposed application. Second, our ABMs have a number of simplifying assumptions, which suggest that the present results are an upper bound on the GEE performance in actual data. These simplifying assumptions include the absence of random error, in either the weight gain process or in the measurement process and complete follow-up with no missing data. Likewise, real data sets display more heterogeneity in rates of growth and the distributions from which parameters are chosen. In principle these could be straightforwardly added to the underlying ABM to test sensitivity when such complications were prominent in any particular application. Finally, we used the reported significance because there is no generally accepted adjustment for the significance, and such corrections were unavailable to us, however future work should test these adjustments [23].

## Conclusions

In sum, then, our results demonstrate that GEE-based approaches to detecting network influence in longitudinal data appear to be relatively robust to homophily on both observed and unobserved characteristics. However, these approaches are unable to distinguish situations in which homophily is present from where it is absent. An agent-based modeling approach allows rapidly one to generate test populations that may exhibit particular forms of confounding or other threats to identification, and empirically verify the extent to which a regression strategy is or is not susceptible to such a postulated threat.

## Additional files

Additional file 1: Agent-Based Model Code. (DOC 29 kb)
Additional file 2: Stata 10 Analytic Code. (DOC 29 kb)

## Abbreviations

ABM: Agent-based model
GEE: Generalized estimating equation
iSPOC: Scientific Papers with Open Communication
