# Supplementary material for: Can longitudinal generalized estimating equation models distinguish network influence and homophily? An agent-based modeling approach to measurement characteristics
Source: BMC Med Res Methodol. 2016 Dec 28;16:174. doi: 10.1186/s12874-016-0274-4 (PMC5192582; doi:10.1186/s12874-016-0274-4)
Supplement: Additional file 1: — Agent-Based Model Code. (DOC 29 kb) [file 12874_2016_274_MOESM1_ESM.doc]

**Additional file 1: Agent-Based Model Code**

The ObeseChild agent-based model was written in Java 1.6 using the Repast Simphony 1.2 libraries and framework. Model documentation, including javadoc and code-base may be found here: <http://dx.doi.org/10.5061/dryad.v3s0k>.

ObeseChild ABM Pseudo-code:

BUILD MODEL:

initialize agents:

set weight

set intrinsicWeightGain

set weightGainStrategy

set weightDifferenceIntolerance

set intrinsicGainDifferenceIntolerance

set weightImportance

initialize network edges:

for each ego:

if random network:

ego chooses n random alters

if weight-based network:

for each potential alter

ego calculates weightDistance

ego determines utilityDistance

ego chooses n friends: weighted-random selection

if intrinsicGain-based network: (this is latent homophily)

for each potential alter

ego calculates intrinsicWeightGainDistance

ego determines utilityDistance

ego chooses n friends: weighted-random selection

      if preferential attachment network

        for each potential alter

          ego calculates distance list based on number of admirers

          ego chooses n friends: weighted-random selection

      if weighted preferential attachment

        for each potential alter

          ego calculates weightDistance

          ego determines utilityDistance

          ego creates distance list from the sum of utilityDistance

and number of admirers

          ego chooses n friends: weighted-random selection

network = set of ego-alter pairs

RUN MODEL:

while step number < runLength:

if dynamic network and step number > 0:

initialize network edges

agents gain weight:

for each ego:

if random gain:

agent weight =+ assigned intrinsicWeightGain

if weight-based gain:

agent weight =+ mean(weight of alters) * multiplier

if intrinsicGain-based gain:

agent weight =+ mean(intrinsic gain of alters)/divisor
