# Supplementary material for: Can longitudinal generalized estimating equation models distinguish network influence and homophily? An agent-based modeling approach to measurement characteristics
Source: BMC Med Res Methodol. 2016 Dec 28;16:174. doi: 10.1186/s12874-016-0274-4 (PMC5192582; doi:10.1186/s12874-016-0274-4)
Supplement: Additional file 2: — Stata 10 Analytic Code. (DOC 29 kb) [file 12874_2016_274_MOESM2_ESM.doc]

**Additional file 2: Stata 10 Analytic Code**

This code repeatedly reads in a dataset produced by the ABM, carries out the GEE-based analysis (using xtgee), and then compiles the results in an spreadsheet for easy post-processing.

*** initialize a local variable with the various condition names**

*** used from the generating ABM**

*** Here: S__ indicates a static network run, D__ indicates a dynamic network run**

*** _R_ indicates that weight gain is Random with respect to the network**

*** _W_ indicates that weight gain is influenced by the observed weight of network partners**

*** __R indicates that network formation is random relative to agent characteristics**

*** __W indicates that network formation displays homophily on observed weight**

*** __A indicates that network formation displays homophily on intrinsic weight gain**

**local combo "sra srr srw swa swr sww"**

**foreach x of local combo {**

**display "combo=`x'"**

**cd "homedirectory\Run_`x'_20110222\output_`x'_20110222"**

**clear**

**set more off**

**set obs 1**

**gen str6 dataused=""**

**save model1_w_wave_pvalues_`x'.dta, replace**

**quietly {**

**forvalues i=0(1)999 {**

**use `x'`i'.dta, clear**

**xtset id tick**

**xi: xtgee egoweight i.tick friendweight friendwt_tm1 egowt_tm1, family(gaussian) link(identity) corr(exchangeable) robust**

*** store the betas in a matrix**

**matrix betas=e(b)**

**matrix list betas**

*** create a vector of variance of the betas from the diagonal of the variance-covariance matrix**

**matrix vars=vecdiag(e(V))**

**matrix list vars**

*** create a diagonal matrix containing the variance estimates only.**

**matrix varsmat=diag(vars)**

**matrix list varsmat**

*** Perform the cholesky decomposition to get the standard error of the betas**

**matrix sesmat=cholesky(varsmat)**

**matrix list sesmat**

*** create a vector of the standard errors**

**matrix sesvec=vecdiag(sesmat)**

**matrix list sesvec**

**/* Initialize matrices for the z-score and p-values to be computed */**

**matrix z=J(1,8,0)**

**matrix p=J(1,8,0)**

**forvalues j=1(1)8 {**

**matrix z[1,`j']=betas[1,`j']/sesvec[1,`j']**

**matrix p[1,`j']=2*(1-normal(abs(z[1,`j'])))**

**}**

*** convert the p-value vector into variables for outputing into the p-value dataset**

**svmat p, name(pvalues)**

**rename pvalues1 p_tick48**

**rename pvalues2 p_tick72**

**rename pvalues3 p_tick96**

**rename pvalues4 p_tick120**

**rename pvalues5 p_altwt**

**rename pvalues6 p_altwt_tm1**

**rename pvalues7 p_egowt_tm1**

**rename pvalues8 p_intercept**

*** convert the beta vector into variables for outputing into the p-value dataset**

**svmat betas, name(betas)**

**rename betas1 b_tick48**

**rename betas2 b_tick72**

**rename betas3 b_tick96**

**rename betas4 b_tick120**

**rename betas5 b_altwt**

**rename betas6 b_altwt_tm1**

**rename betas7 b_egowt_tm1**

**rename betas8 b_intercept**

*** convert the SE for beta vector into variables for outputing into the p-value dataset**

**svmat sesvec, name(se)**

**rename se1 se_tick48**

**rename se2 se_tick72**

**rename se3 se_tick96**

**rename se4 se_tick120**

**rename se5 se_altwt**

**rename se6 se_altwt_tm1**

**rename se7 se_egowt_tm1**

**rename se8 se_intercept**

**gen str6 dataused="`x'`i'"**

**gen datanum=substr(dataused,4,6)**

**destring datanum,replace**

**keep dataused datanum b_* se_* p_***

**order dataused datanum b_* se_* p_***

**duplicates drop**

**keep if _n==1**

**append using model1_w_wave_pvalues_`x'**

**sort datanum**

**save model1_w_wave_pvalues_`x', replace**

**}**

**}**

**outsheet using modl1_w_wave_pvalues_`x'.csv,comma replace**

**}**
